# Author Correction: Single-cell analysis of EphA clustering phenotypes to probe cancer cell heterogeneity

**DOI:** 10.1038/s42003-020-01238-z

**Published:** 2020-09-07

**Authors:** Andrea Ravasio, Myint Z. Myaing, Shumei Chia, Aditya Arora, Aneesh Sathe, Elaine Yiqun Cao, Cristina Bertocchi, Ankur Sharma, Bakya Arasi, Vin Yee Chung, Adrianne C. Green, Tuan Zea Tan, Zhongwen Chen, Hui Ting Ong, N. Gopalakrishna Iyer, Ruby YunJu Huang, Ramanuj DasGupta, Jay T. Groves, Virgile Viasnoff

**Affiliations:** 1 National University of Singapore, Singapore, Singapore; 2 Pontificia Universidad Católica de Chile, Santiago, Chile; 3 Genome Institute of Singapore, ASTAR, Singapore, Singapore; 4 National Cancer Centre Singapore, Singapore, Singapore; 5 Cancer Science Institute of Singapore, National University of Singapore, Singapore, Singapore; 6 School of Medicine & Graduate Institute of Oncology, College of Medicine, National Taiwan, Taipei, Taiwan; 7 University Department of Obstetrics & Gynaecology, Yong Loo Lin School of Medicine, National University of Singapore, Singapore, Singapore; 8 University of California, Berkeley, CA USA; 9 Centre National de la Recherche Scientifique, Singapore, Singapore

Correction to: *Communications Biology* 10.1038/s42003-020-01136-4, published online 6 August 2020.

The original version of this Article contained an error in the affiliation of the author Tuan Zea Tan, which was incorrectly given as National University Health System, Singapore, Singapore. This has now been corrected to Cancer Science Institute of Singapore, National University of Singapore, Singapore in both the PDF and HTML versions of the Article.

