# Author Correction: Single-cell analysis of EphA clustering phenotypes to probe cancer cell heterogeneity

**DOI:** 10.1038/s42003-020-01395-1

**Published:** 2020-11-06

**Authors:** Andrea Ravasio, Myint Z. Myaing, Shumei Chia, Aditya Arora, Aneesh Sathe, Elaine Yiqun Cao, Cristina Bertocchi, Ankur Sharma, Bakya Arasi, Vin Yee Chung, Adrienne C. Greene, Tuan Zea Tan, Zhongwen Chen, Hui Ting Ong, N. Gopalakrishna Iyer, Ruby YunJu Huang, Ramanuj DasGupta, Jay T. Groves, Virgile Viasnoff

**Affiliations:** 1 National University of Singapore, Singapore, Singapore; 2 Pontificia Universidad Católica de Chile, Santiago, Chile; 3 Genome Institute of Singapore, A­STAR, Singapore, Singapore; 4 University of California, Berkeley, CA USA; 5 Cancer Science Institute of Singapore, National University of Singapore, Singapore, Singapore; 6 National Cancer Centre Singapore, Singapore, Singapore; 7 School of Medicine & Graduate Institute of Oncology, College of Medicine, National Taiwan, Taiwan; 8 University Department of Obstetrics & Gynaecology, Yong Loo Lin School of Medicine, National University of Singapore, Singapore, Singapore; 9 Centre National de la Recherche Scientifique, Singapore, Singapore

Correction to: *Communications Biology* 10.1038/s42003-020-01136-4, published online 6 August 2020.

In the original published version of the Article, contributing author Adrienne C. Greene of the University of California, Berkeley was incorrectly listed as Adrianne C. Green of the National Cancer Centre Singapore. The error has been corrected in the HTML and PDF versions of the Article.

