# Supplementary material for: Single-cell analysis of EphA clustering phenotypes to probe cancer cell heterogeneity
Source: Commun Biol. 2020 Aug 6;3:429. doi: 10.1038/s42003-020-01136-4 (PMC7411022; doi:10.1038/s42003-020-01136-4)
Supplement: Supplementary file 5 — Description of Additional Supplementary Files [file 42003_2020_1136_MOESM5_ESM.pdf]

Description of Additional Supplementary Files

File Name: **Supplementary Movie 1**
Description: Time-lapse of EphA cluster formations in PEO1 cells. Left, fluorescence images of Alexa568-ephrin A1. Ligand has free later diffusibility on the supported bilayer. Thus, dynamics of clustering are to be attributed to active transport of the EphA receptor by the cell cytoskeleton. Middle, Reflection Interference Contrast Microscopy (RICM) images. Dark regions (destructive interference) denote the location where cell membrane is intimate contact with the supported bilayer. Importantly, these regions correspond to the highest fluorescence on the left images. Right, phase contrast images of the cells. Movies length = 30 min. Frame rate = 30 seconds/frame.

File Name: **Supplementary Movie 2**
Description: Time-lapse of EphA cluster formations in HeyA8 cells. Left, fluorescence images of Alexa568-ephrin A1. Ligand has free later diffusibility on the supported bilayer. Thus, dynamics of clustering are to be attributed to active transport of the EphA receptor by the cell cytoskeleton. Middle, Reflection Interference Contrast Microscopy (RICM) images. Dark regions (destructive interference) denote the location where cell membrane is intimate contact with the supported bilayer. Importantly, these regions correspond to the highest fluorescence on the left images. Right, phase contrast images of the cells. Movies length = 30 min. Frame rate = 30 seconds/frame.

File Name: **Supplementary Data 1**
Description: Raw data for all graph figures.
