# Supplementary material for: Single-cell analysis of EphA clustering phenotypes to probe cancer cell heterogeneity
Source: Commun Biol. 2020 Aug 6;3:429. doi: 10.1038/s42003-020-01136-4 (PMC7411022; doi:10.1038/s42003-020-01136-4)
Supplement: Supplementary file 6 — Reporting Summary [file 42003_2020_1136_MOESM6_ESM.pdf]

nature research

Corresponding author(s): Virgile Viasnoff

Last updated by author(s): Jun 29, 2020

Reporting Summary

Nature Research wishes to improve the reproducibility of the work that we publish. This form provides structure for consistency and transparency in reporting. For further information on Nature Research policies, see Authors & Referees and the Editorial Policy Checklist.

Statistics

For all statistical analyses, confirm that the following items are present in the figure legend, table legend, main text, or Methods section.

n/a	Confirmed	
☐	☒	The exact sample size (n) for each experimental group/condition, given as a discrete number and unit of measurement
☐	☒	A statement on whether measurements were taken from distinct samples or whether the same sample was measured repeatedly
☐	☒	The statistical test(s) used AND whether they are one- or two-sided<br>*Only common tests should be described solely by name; describe more complex techniques in the Methods section.*
☐	☒	A description of all covariates tested
☐	☒	A description of any assumptions or corrections, such as tests of normality and adjustment for multiple comparisons
☐	☒	A full description of the statistical parameters including central tendency (e.g. means) or other basic estimates (e.g. regression coefficient) AND variation (e.g. standard deviation) or associated estimates of uncertainty (e.g. confidence intervals)
☐	☒	For null hypothesis testing, the test statistic (e.g. F, t, r) with confidence intervals, effect sizes, degrees of freedom and P value noted<br>*Give P values as exact values whenever suitable.*
☒	☐	For Bayesian analysis, information on the choice of priors and Markov chain Monte Carlo settings
☒	☐	For hierarchical and complex designs, identification of the appropriate level for tests and full reporting of outcomes
☐	☒	Estimates of effect sizes (e.g. Cohen's d, Pearson's r), indicating how they were calculated

Our web collection on statistics for biologists contains articles on many of the points above.

Software and code

Policy information about availability of computer code

Data collection	Microscopy data have been collected with MetaMorph.
Data analysis	Custom made MatLab code for image analysis is available from the corresponding author upon reasonable request. Python codes used for image feature extraction and unbiased machine learning method are available at Github: https://github.com/aneeshsathe/Ephrin_cluster_analysis. Data analysis, statistics and presentation have been made using GraphPad Prism 6 Version 6.01

For manuscripts utilizing custom algorithms or software that are central to the research but not yet described in published literature, software must be made available to editors/reviewers. We strongly encourage code deposition in a community repository (e.g. GitHub). See the Nature Research guidelines for submitting code & software for further information.

Data

Policy information about availability of data

All manuscripts must include a data availability statement. This statement should provide the following information, where applicable:

- Accession codes, unique identifiers, or web links for publicly available datasets
- A list of figures that have associated raw data
- A description of any restrictions on data availability

Previously generated single cell transcriptomics data from patient-derived cell lines are available in GEO, accession number GSE117872. All original images, data and MATLAB codes are available from the corresponding author upon reasonable request. All the data are currently stored in data storage facility at Mechanobiology Institute, SingaporeAvailability statement for images, data and codes can be found in the method section "Data and resources availability".

Field-specific reporting

Please select the one below that is the best fit for your research. If you are not sure, read the appropriate sections before making your selection.

☒ Life sciences ☐ Behavioural & social sciences ☐ Ecological, evolutionary & environmental sciences

For a reference copy of the document with all sections, see nature.com/documents/nr-reporting-summary-flat.pdf

Life sciences study design

All studies must disclose on these points even when the disclosure is negative.

Sample size	Each distribution is based on N>200 single-cell analysis to best describe full population distribution. For clonal colonies all cells retrieved have been analyzed and reported.
Data exclusions	Rational for data exclusion criteria is described in Fig. 3 "Reproducible measurement of SEphA scores." and in the results section "Scoring cluster morphologies ". Data have been excluded only if analysis of internal control sample revealed defective preparation.
Replication	Each experiment report single cell analysis collected from at least three independent experiments. Rational for data normalization is described in Fig. 3 "Reproducible measurement of SEphA scores." and in the results section "Scoring cluster morphologies ".
Randomization	N/A
Blinding	Image data collection and analysis have been performed by distinct researchers.

Reporting for specific materials, systems and methods

We require information from authors about some types of materials, experimental systems and methods used in many studies. Here, indicate whether each material, system or method listed is relevant to your study. If you are not sure if a list item applies to your research, read the appropriate section before selecting a response.

Materials & experimental systems

n/a	Involved in the study	
☐	☒	Antibodies
☐	☒	Eukaryotic cell lines
☒	☐	Palaeontology
☒	☐	Animals and other organisms
☒	☐	Human research participants
☒	☐	Clinical data

Methods

n/a	Involved in the study	
☒	☐	ChIP-seq
☒	☐	Flow cytometry
☒	☐	MRI-based neuroimaging

Antibodies

Antibodies used	Anti EphA2 (Cell Signaling, cat no. 6997, DIlution 1:1000)<br>Anti-beta actin (Pierce, cat. no. MA515739, Dilution 1:5000)<br>Anti-rabbit HRP (Invitrogen, cat. no. G21234, Dilution 1:5000)<br>anti-mouse HRP (Invitrogen, cat. no. G21040, Dilution 1:10000)
Validation	All antibodies used are commercially available and have been chosen based on previous report from literature.

Eukaryotic cell lines

Policy information about cell lines

Cell line source(s)	The lung, ovarian, gastric and breast cancer cell lines were obtained commercially from ATCC or were kindly donated by Dr. Ken Yamaguchi from Kyoto University Graduate School of Medicine
Authentication	The identities of the cell lines were checked comparing the STR profile (Indexx BioResearch) of each cell line to its original tumor.
Mycoplasma contamination	All the cells used in this study were tested for mycoplasma using Mycoprobe Mycoplasma Detection kit from R&D system as described in Method session "Cell culture, dissociation, seeding and fixation".
Commonly misidentified lines (See ICLAC register)	MKN28. Cell line and possible contaminating cells are of the same origin.
